# Phlorotannins from *Fucus vesiculosus*: Modulation of Inflammatory Response by Blocking NF-κB Signaling Pathway

**DOI:** 10.3390/ijms21186897

**Published:** 2020-09-20

**Authors:** Marcelo D. Catarino, Ana Silva, Maria T. Cruz, Nuno Mateus, Artur M.S. Silva, Susana M. Cardoso

**Affiliations:** 1 LAQV-REQUIMTE, Department of Chemistry, University of Aveiro, 3810-193 Aveiro, Portugal; mcatarino@ua.pt (M.D.C.); artur.silva@ua.pt (A.M.S.S.); 2 CNC.IBILI, Faculty of Pharmacy, University of Coimbra, Health Sciences Campus, 3000-548 Coimbra, Portugal; anacrs@cnc.uc.pt (A.S.); trosete@ff.uc.pt (M.T.C.); 3 REQUIMTE/LAQV, Department of Chemistry and Biochemistry, Faculty of Sciences, University of Porto, 4169-007 Porto, Portugal; nbmateus@fc.up.pt

**Keywords:** brown algae, *Fucus vesiculosus*, marine bioactives, phenolic compounds, phlorotannins, anti-inflammatory, antioxidant, Raw 264.7

## Abstract

Due to their large spectrum of bioactive properties, much attention has recently been drawn to phlorotannins—i.e., phenolic compounds characteristic from brown macroalgae. This study aimed to evaluate the antioxidant and anti-inflammatory properties of *F. vesiculosus* phlorotannin extracts and purified fractions. Overall, the crude extract and its ethyl acetate fraction (EtOAc) showed good radical scavenging activity, particularly towards nitric oxide (NO^•^). Subsequent subfractions of EtOAc (F1 to F9) with different molecular weights were then shown to inhibit lipopolysaccharide-induced NO^•^ production in macrophages, with stronger effects being observed for fractions of lower MWs. Of the three intracellular markers analyzed, inducible NO^•^ synthase showed the highest sensitivity to almost all the phlorotannin-rich samples, followed by interleukin 1β and cyclooxygenase 2, which was only inhibited by F2. Furthermore, this subfraction inhibited the phosphorylation and degradation of inhibitory protein κBα, thus preventing the activation of NF-κB and blocking the inflammatory cascade at the transcriptional level. This sample was characterized by the presence of a major compound with a deprotonated molecular ion at *m/z* 507 with a fragmentation pattern coherent with that of a phlorotannin derivative. Overall, this work unveiled some of the mechanistic aspects behind the anti-inflammatory capacity of phlorotannins from *F. vesiculosus*, endorsing its use as a possible natural source of anti-inflammatory compounds.

## 1. Introduction

*Fucus vesiculosus*, commonly known as bladder wrack, is a species of brown seaweed growing in cold-temperate waters, largely distributed around the North Atlantic shores, extending from the White Sea to the south of the Canary Islands at the east, and from south Greenland to North Carolina at the west, also occurring to a lesser extent along the Northeast Pacific shores, extending from Alaska to California [1]. The consumption of this seaweed has reputed health benefits against several diseases, being most commonly used for the treatment of goiter and thyroid-related complications due to its high concentrations of iodine. In addition, *F. vesiculosus* is a wealthy source of a range of bioactive compounds, including fucoidan, fucoxanthin, phlorotannins and several vitamins, all presenting a broad spectrum of bioactivities with great potential for application in the food, pharmaceutical and cosmetic industries [2]. Of these, phlorotannins—i.e., phenolic compounds characteristic from brown algae—have drawn much attention in the recent years due to their potential health benefits and pharmacological applications. These phenolic compounds are produced in seaweeds through the C–C and/or C–O–C oxidative coupling of phloroglucinol (1,3,5-trihydroxybenzene) monomeric units forming structures that may range from simple molecules of 126 Da to very large and complex polymers, being very susceptible to high variability depending on the seaweed species, environmental conditions, seasonality, and several other factors [3]. According to the nature of the structural linkages between phloroglucinol units, they can be characterized into four different subclasses: phlorethols (possessing an ether linkage), fucols (possessing C-C linkage), fucophlorethols (possessing either ether and C-C linkages) and eckols (possessing a dibenzodioxin linkage). Within the subclass of phlorethols there are also the fuhalols which are ether-linked phlorotannins that possess at least one additional hydroxyl group. Likewise, eckols with additional hydroxyl groups are known as carmalols [4]. The procedures used for phlorotannins extraction are widely variable and include aqueous mixtures of methanol, ethanol, acetone, and more recently deep eutectic solvents, as well as different extraction techniques including microwave, ultrasounds, enzymes, pressurized liquids and others [5,6,7,8,9,10,11]. Acetone is believed to inhibit the interactions occurring between phlorotannins and proteins, breaking the hydrogen bonds between phlorotannin–protein complexes during extraction, causing an increase in the total yield of extraction [12]. According to Koivikko et al. [13], the most suitable solvent for phlorotannins extraction is a mixture acetone: H_2_O (7:3) which is in agreement with our previous results [14]. In order to obtain a phlorotannins rich extract, the mixture resulting from acetone:H_2_O extraction can be purified using different strategies. One of the most common approach is through solvent partitioning and gel filtration with Sephadex LH-20, although other works have tried cellulose filtration, ultra-filtration, dialysis and others [15,16,17]. The different affinities of these compounds to the solvent and the Sephadex LH-20 gel allows their separation and subsequent purification from the other extracted compounds.

The high variability of phlorotannins grants them a large spectrum of bioactivities, which include antioxidant, antimicrobial, antiviral, antitumor, antidiabetic, anti-inflammatory and many other bioactivities. In particular, their anti-inflammatory potential has attracted the interest of several authors that have already proven that phlorotannins from different species, including *Ecklonia cava*, *Ecklonia stolonifera* and *Eisenia bicyclis*, mainly rich in eckols, have the capacity to interfere in the inflammatory process at different levels [18,19,20]. 

Inflammation is the central feature of many pathophysiological conditions in response to tissue injury and as part of host defense against microorganisms. During this process, the first line of defense is provided by the macrophages which, in the presence of a stimulus, such as microbial lipopolysaccharide (LPS), interferon-γ, tumor necrosis factor (TNF) or oxidative stress, produce several pro-inflammatory mediators, including reactive oxygen and nitrogen species, such as nitric oxide radical (NO^•^) and superoxide radical anion (O_2_^•−^), prostaglandins, cytokines, including TNF-α, interleukin (IL)-1β, IL-6, and others [21]. Under normal conditions, the release of such markers is of utmost importance, manifesting rapidly and severely upon injury, but persisting only for a short period of time until the resolution of the harmful stimuli. However, the abnormal production of these markers might result in damages to the host tissue itself, leading to a vicious circle that, if maintained over a long period of time, may evolve to chronic inflammation-related illnesses, such as inflammatory bowel diseases or rheumatoid arthritis [22].

The regulation of such pro-inflammatory molecules is mediated by several transcription factors. Of these, NF-κB is perhaps one of the most important regulators of the innate immunity, being responsible for the transcription of various genes involved in the inflammatory signaling cascades [23]. Under normal conditions, NF-κB is located in the cytoplasm bound to inhibitory proteins κB (IκB), which exist in the forms α, β and ε, the former being the most well studied [24]. Upon a stimulation, such as LPS, an intracellular signaling cascade triggers the phosphorylation of the IκBα which is subsequently ubiquitinated and degraded by the proteasome, releasing the NF-κB that translocates into the nucleus and binds to the DNA specific binding site and enhancer regions and induces the transcription of several pro-inflammatory genes that encode for the biochemical mediators involved in the inflammatory process [25]. Therefore, the inhibition of NF-κB transcriptional activity as well as its down-stream molecules may represent a viable therapeutic strategy for intervention in pathologies presenting a strong inflammatory component. In this context, the knowledge of phytochemicals’ molecular mechanisms became a good strategy in the search for novel anti-inflammatory compounds, and phlorotannins are emerging as potential candidates capable of modulating inflammation through the inhibition of the expression and/or activity of multiple inflammatory markers [26,27,28]. The existing literature on the potential anti-inflammatory capacity of *F. vesiculosus-*derived phlorotannins, which correspond mainly to fucols and fucophlorethols, remains very scarce, and the few studies carried out on this topic were only focused on their capacity to inhibit the release of NO^•^ in LPS or phorbol myristate acetate (PMA)-stimulated macrophages [29,30,31]. Thus, this work aimed to explore the molecular mechanisms and signaling pathways involved in the anti-inflammatory properties of *F. vesiculosus* extract and further phlorotannin purified fractions. Additionally, the characterization of the sample with the strongest anti-inflammatory activity was investigated in an attempt to unveil the possible compounds responsible for such effects.

## 2. Results and Discussion

### 2.1. Antioxidant Properties of Fucus Vesiculosus Crude Extract (CRD) and Phlorotannin-Enriched Fraction (EtOAc)

The antioxidant activities of *F. vesiculosus* CRD and EtOAc were screened for their ability to scavenge different free radicals, namely peroxyl (RCOO^•^), superoxide anion (O_2_^•−^) and nitric oxide (NO^•^), as well as to inhibit the activity of xanthine oxidase, which is known to catalyze the oxidation of hypoxanthine and xanthine to uric acid with the concomitant production of O_2_^•−^, making this enzymatic system an important biological source of ROS with key roles in several pathogenic processes.

Overall, for each assay, a dose-dependent activity was observed in crude and EtOAc samples (data not shown) and the corresponding IC_50_ values are shown in Table 1. Interestingly, even though the EtOAc had higher concentration in phlorotannins, the best radical scavenging activity was observed for the CRD, particularly in O_2_^•−^ and NO^•^, for which the IC_50_ values were two and three times stronger, suggesting that, in addition to phlorotannins, the CRD might contain other compounds with scavenging activity, such as fucoxanthin or tocopherols that are possibly contributing to these results. On that basis, since the EtOAc is a phlorotannin purified fraction resultant from solvent partitioning of the CRD, it lacks several compounds that were retained in the other fractions, and therefore the possible synergistic effects were lost, causing a decrease in the scavenging activity of the EtOAc. Nevertheless, it is worth noting that the IC_50_ values of the samples against NO^•^ were comparable or even lower than that of the standard compound, which is a particularly relevant result since this free radical plays a pivotal role in the signaling and pathogenesis of inflammation and inflammation-related diseases, thus constituting a potential target for developing anti-inflammatory therapeutics [32]. Therefore, the good scavenging capacity demonstrated by these samples against NO^•^ suggests that they may also display a promising anti-inflammatory potential.

Interestingly, the antioxidant potential of the samples herein studied, considering the values observed for oxygen radical absorbance capacity (ORAC), O_2_^•−^ and NO^•^, are far more promising than those reported in previous studies on different *Fucus* species. For instance, in the work of Wang and co-workers [33], among 24 extracts of 12 different seaweeds, the 70% acetone extract of *F. vesiculosus* exhibited the highest ORAC value of 2567 μmol TE/g extract which is still considerably below compared to the results herein observed. It is also worth noting that, although these results fall short compared to a methanol extracts of rosemary, turmeric or cloves (4360, 5440 and 6150 μmol TE/g extract, respectively) [34], they are still comparable and even higher than the ORAC values reported for some of the highest antioxidant fruits and spices, such as cinnamon (3130 μmol TE/g extract, methanol) [34], strawberry (540 μmol TE/g extract, acetone 50%) [35] or coffee (2463–2828 μmol TE/g extract, water) [36]. Regarding the O_2_^•−^ scavenging capacity, the *F. vesiculosus* crude extract produced in this work revealed approximately two- and seven-times stronger activity compared to those previously reported for 30% and 70% ethanol extracts of the same species, respectively [31]. In turn, in other work, a purified phlorotannin extract from *F. vesiculosus* was not able to achieve 10% of O_2_^•−^ scavenging for a concentration of 5 mg/mL [37]. 

According to Lopes et al. [30], the scavenging of 50% of the sodium nitroprusside (SNP)-generated NO^•^ was achieved only for concentrations between 2 and 4 mg/mL of a purified fraction of *F. spiralis*. More recently, a similar study reported that, among four different *Fucus* species, the phlorotannin-purified extracts of wild and cultivated *F. vesiculosus* showed IC_50_ values of 1330.6 and 2072.3 μg/mL, respectively, while the highest NO^•^ scavenging capacity was achieved with *F. guiryi* purified extract, which presented an IC_50_ of 451.9 μg/mL, approximately two- and six-times higher compared to those of the EtOAc and CRD herein studied, respectively.

It is well known that the phlorotannin profile and content in seaweeds are very susceptible to variability, depending on several factors, such as environmental conditions, geographical origin, harvest season, post-harvest processing and others. This variability could explain the differences between the presented results and other previous studies. Other possible explanation is that *Fucus vesiculosus* and *Ascophyllum nodosum* are usually blended in the wild, so they are commonly harvested and processed together. The extraction of phlorotannins can therefore result in a mix of both species, which may impact on the biological properties of the extracts [2].

In turn, the inhibitory effect of EtOAc against xanthine oxidase (XO) was considerably higher compared to that of the CRD, with the former showing an activity two-times higher than the latter, indicating that, in this case, phlorotannins might be important contributors for the inhibitory effects observed. While the effects and structure-relation between flavonoids and several other terrestrial phenolics with XO are well established [38,39], very few authors have reported the effects of phlorotannins towards this enzyme. Notwithstanding, evidences point that, similar to the terrestrial phenolics, the XO inhibitory capacity seems to be positively correlated with the amount of phlorotannins in the samples, since, according to the work Tanniou et al. [40], from the five different *S. muticum* sampled along the European coast, the highest XO inhibition was observed for those with the highest content in phlorotannins. Likewise, a recent study described that among four different species of *Fucus*, the extract from *F. serratus*, which had the highest concentration of phlorotannins (2.76 ± 0.20 μg phloroglucinol equivalentsmg dry extract), exhibited the best inhibitory capacity against XO, followed by *F. guiryi* (2.15 ± 0.22 μg PGE/mg DE), *F. spiralis* (1.56 ± 0.10 μg PGE/mg DE) and *F. vesiculosus* (1.45 ± 0.10 μg PGE/mg DE), thus supporting the evidence that the phlorotannin content of the extracts is indeed correlated with their inhibitory capacity towards XO [37]. Interestingly, although the IC_50_ values obtained for the CRD and EtOAc herein studied were considerably higher compared to allopurinol (approximately 30 and 10 times, respectively), their inhibitory capacity are much stronger compared to that described by Lopes et al. [37] who reported IC_50_ values more than 310 times that of allopurinol for *F. vesiculosus* phlorotannin purified extract and of 60–300 times higher for extracts of other *Fucus* species. Such differences might be related to variations in the harvesting season and location, and extraction procedures.

### 2.2. Anti-Inflammatory Properties of Fucus Vesiculosus CRD and Its Fractions in Raw 264.7 Cells

#### 2.2.1. Effects on Cell Viability and LPS-Induced NO^•^ Production

**Figure 1:**
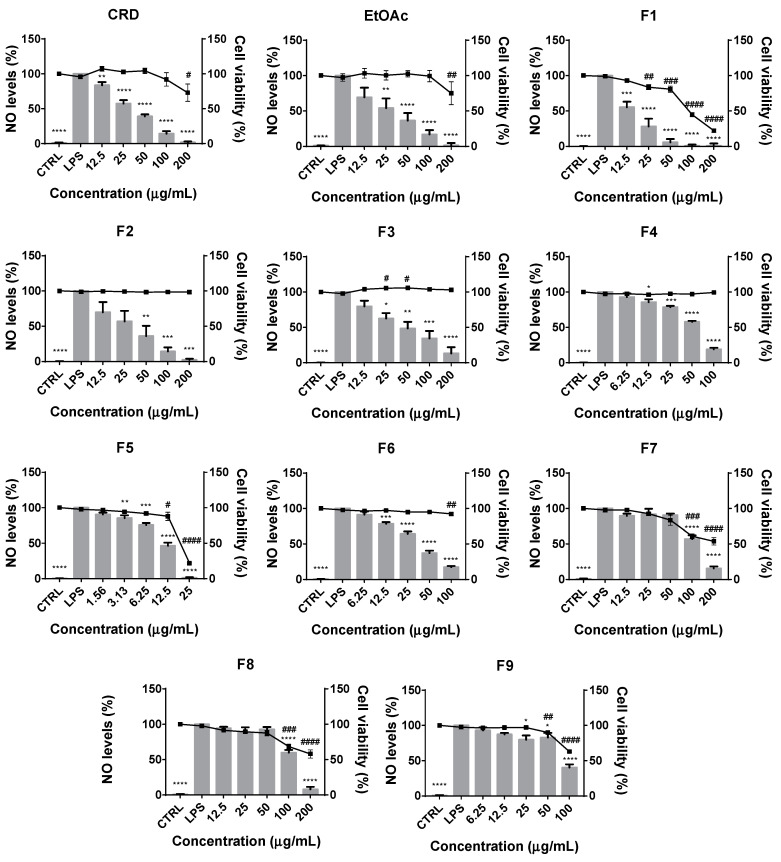
Effects of *F. vesiculosus* crude extract (CRD), ethyl acetate fraction (EtOAc) and subsequent subfractions (F1–F9) on the NO^•^ production (grey bars) and viability (■) of LPS-stimulated Raw 264.7 cells. Data represent the mean ± SEM from at least 3 independent experiments. * *p* < 0.05, ** *p* < 0.01, *** *p* < 0.001 and **** *p* < 0.0001, indicate that NO^•^ production is significantly different from the positive control (with LPS) and ^#^ *p* < 0.05, ^##^ *p* < 0.01, ^###^ *p* < 0.001 and ^####^ *p* < 0.0001 indicate that cells viability are statistically different from the negative control (CTRL, without LPS), as determined by one-way ANOVA followed by Dunnet’s post hoc test.

Since *F. vesiculosus* phlorotannins extracts displayed good NO^•^ scavenging capacity, the next step was to evaluate whether these compounds could effectively display anti-inflammatory capacity in a biological system of inflammation—i.e., in Raw 264.7 cells stimulated with the *Toll*-like receptor 4 (TLR-4) agonist, lipopolysaccharide (LPS). As expected, control cells (untreated) produced very low NO^•^ levels, while the culture medium of the cells treated with the LPS for 24 h showed a markedly elevated NO^•^ content (Figure 1). As shown in Figure 1, both crude and EtOAc samples (100 μg/mL) inhibited the LPS-induced NO^•^ production in a dose-dependent manner, decreasing the NO^•^ production to 14.08 ± 3.68 and 16.80 ± 6.32%, respectively, comparing with the LPS. However, at 200 μg/mL a decrease in cell viability indicates a cytotoxic effect.

These results are in line with those reported by Zaragoza and co-workers [31], who demonstrated that a phlorotannin-rich extract from *F. vesiculosus* (30–35% ethanol) could effectively inhibit the production of NO^•^ by LPS-exposed macrophages, although their extracts only achieved 50% of inhibition for a concentration of 95 μg/mL. Notably, the activities herein observed for the *F. vesiculosus* CRD and EtOAc are also much stronger than those reported by Barbosa et al. [29] for a purified extract of phlorotannins obtained from cultivated *F. vesiculosus*, which at 317 μg/mL only achieved 25% of inhibition. Other *Fucus* species assayed in the same work exhibited more promising IC_25_ values with *F. guirii* and *F. spiralis*, showing identical activity (IC_25_ = 97.7 and 95.9 μg/mL), followed by *F. serratus* (IC_25_ = 77.0 μg/mL) and wild *F. vesiculosus* (IC_25_ = 56.5 μg/mL) harvested in the north coast of Portugal. In any case, inhibitions of 50% or close were only achieved at the concentration of 500 μg/mL for all the samples.

In an attempt to further explore possible relations between the phlorotannins’ molecular sizes and their anti-inflammatory effects, fractionation of EtOAc was carried out in a Sephadex LH-20 gel following the procedure previously described by Wang et al. [15] which, as demonstrated by the authors, allows for the separation of compounds into fractions of crescent molecular weights, based on hydrogen-bonding between the phenolic OH groups with the matrix and a proper eluent system. According to Figure 1, it is noticeable that phlorotannins with more complex structures (fractions F7, F8 and F9) exhibited neglectable inhibitory effects on LPS-induced NO^•^ production, showing a certain cytotoxic effect at the highest concentrations. In turn, exceptional effects were observed for the fractions F2, F3, F4 and F6, all revealing inhibitions over 80% without affecting cell viability below 90% for any of the concentrations tested. Good results were obtained as well for F1 and F5, although both displayed accentuated toxicity for concentrations above 12.5 μg/mL, particularly F5. Nevertheless, at this concentration, both fractions were able to inhibit NO^•^ production to approximately 50% (compared to LPS positive control) while maintaining the cell viability above 90%, compared to the control untreated cells (CRL).

Barbosa et al. [29] has previously suggested that the qualitative composition of the extracts could be determinant when considering the effect of phlorotannins on the production of NO^•^ in Raw 264.7 exposed to LPS. They observed that samples with very different phlorotannin contents such as *F. guirii* and *F. spiralis* (288.4 and 165.9 μg PGE/100 mg, respectively) displayed identical inhibitory capacities (IC_25_ = 97.7 and 95.9 μg/mL, respectively), while samples with similar phlorotannin contents from cultivated and wild *F. vesiculosus* (110.3 and 144.5 μg PGE/100 mg, respectively), evoke a different effect on NO^•^ production (IC_25_ = 317.7 and 56.5 μg/mL, respectively). Following this logic and considering that our results demonstrate that phlorotannins with higher polymerization degrees and complexity are less active than simpler phlorotannins, it is feasible to hypothesize that the balance between polymeric and oligomeric compounds in a phlorotannin extract may determine its potential to inhibit the production of NO^•^ by LPS-stimulated macrophages.

The involvement of phlorotannins in the inflammatory signaling cascades and, in particular, on the modulation of NO^•^ levels has been already reported by several authors, although there are few studies focusing on the anti-inflammatory activity of phlorotannin extracts from Fucales [41,42,43,44]. In the particular case of phlorotannin extracts from *F. vesiculosus*, the existing studies only focused on the inhibition of NO^•^ release by LPS and/or PMA-stimulated macrophages [29,30,31] and, to the best of our knowledge, this is the first study evidencing a relation between their complexity and anti-inflammatory activity, although further research must be carried out to better understand the structure–activity relation of phlorotannins in inflammation.

#### 2.2.2. Effects on the Expression of iNOS, COX-2 and IL-1β

Upon a pro-inflammatory stimulus, the enzyme iNOS converts L-arginine to L-citrulline with the concomitant release of NO^•^ [38]. Therefore, in addition to the scavenging mechanism, phlorotannins may also interfere with the production of NO^•^ in LPS-stimulated cells by the down-regulation of iNOS expression. In fact, when comparing the results from the previous in chemico and in vitro assays regarding the samples’ activity towards NO^•^, it is notable that, although the CRD exhibited higher NO^•^ scavenging capacity over EtOAc, such discrepancy did not occur in the cellular based system. These observations suggest that phlorotannins might not act solely as NO^•^ scavengers but also as inhibitors of the iNOS activity and/or expression, and even against other inflammatory markers, such as COX-2 or IL-1β.

Therefore, in order to deeply explore the possible involvement of the *F. vesiculosus* phlorotannins in the modulation of key inflammatory proteins expressed by Raw 264.7 in the presence of a pro-inflammatory stimulus, we further performed Western blotting with the samples that showed at least 25% inhibition of LPS-induced NO^•^ production without affecting cell viability below 90% (200 μg/mL of F2 and F3, 100 μg/mL of crude, EtOAc, F4 and F6, and 12.5 μg/mL of F1 and F5).

**Figure 2:**
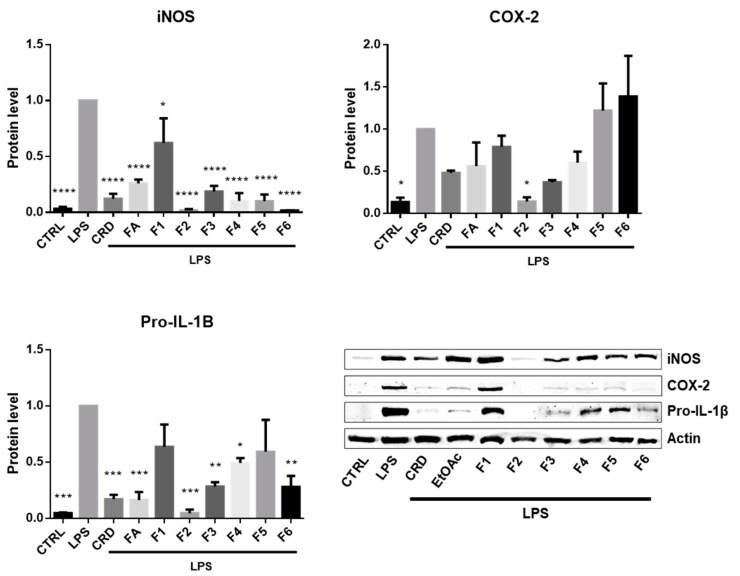
The effects of *F. vesiculosus* extract and partitioned fractions on the expression of pro IL-1β, iNOS and COX-2 in LPS-stimulated Raw 264.7 cells. The immunoblots presented are representative of three independent blots; * *p* < 0.05, ** *p* < 0.01, *** *p* < 0.001 and **** *p* < 0.0001 indicate significant differences from the positive control (LPS), as determined by one-way ANOVA, followed by Dunnett’s post hoc test. CRD—crude extract, EtOAc—ethyl acetate fraction.

As depicted in Figure 2, under normal conditions, the expression of iNOS, COX-2 and IL-1β is very residual, while the stimulation with LPS triggered the expression of these pro-inflammatory proteins, indicating that the cells entered into an inflammatory stage.

Upon the treatment with the different *F. vesiculosus* samples, it was clear that iNOS was the most affected marker. Since this enzyme is the major one responsible for the production of NO^•^ during inflammation, the inhibition of its expression is expected to cause a decrease in the NO^•^ levels produced by LPS-stimulated cells. Therefore, the results observed in Figure 2 for iNOS are in agreement those from Figure 1, indicating that the reduction in NO^•^ release was caused by the down-regulation of the LPS-induced iNOS expression in presence of the *F. vesiculosus* extract and phlorotannin fractions.

Following iNOS, pro-IL-1β was the second most affected marker, being inhibited by almost every sample. This cytokine is also an important marker in the inflammatory response, with a determinant role in the establishment of chronic inflammation and autoimmune diseases [45]. Therefore, targeting this interleukin would be a possible approach to alleviate the severity of chronic inflammation and attenuate autoimmune diseases. Overall, the *F. vesiculosus* extract and phlorotannin fractions markedly inhibited the expression of this interleukin after a pro-inflammatory stimulus, with F2 exhibiting the strongest inhibitory capacity. In turn, F1 and F5 did not exhibit significant inhibition of LPS-stimulated pro-IL-1β expression, although this does not necessarily mean that they do not affect IL-1β release. For the pro-IL-1β to become active it has firstly to be cleaved proteolytically by interleukin-1 converting enzyme (ICE). The inhibition of the ICE activity would therefore block the maturation of pro-IL-1β into active IL-1β and consequently prevent its release and the sustenance of the inflammatory stimulus. This mechanism of action is also described for pralnacasan, a potent orally bioavailable drug that inhibits the maturation of IL-1β by reversibly inhibiting ICE [46]. However, to verify if F1 and F5 could display a similar mechanism of action, further studies are necessary in order to evaluate their effect against ICE.

On the other hand, with exception of F2, none of the samples were able to significantly inhibit the expression COX-2. In fact, cells treated with F5 and F6 revealed a tendentially exacerbated expression of this enzyme, although this does not imply an intensification of the pro-inflammatory response but rather a possible post-translational inhibitory effect that simultaneously favors the accumulation of COX-2 due to the absence of the negative feedback of prostaglandins. This means that, because the synthesis of endogenous prostaglandins is blocked, the cell does not receive the feedback signal to stop expressing the COX-2 thus continuing to accumulate the enzyme. This mechanism of action is actually described for the non-steroidal anti-inflammatory drug indomethacin, a well-known COX inhibitor commercialized under the trade name of Indocin. This drug strongly inhibits the LPS-induced COX-2 activity, decreasing the synthesis of prostaglandins that favor COX-2 degradation and at the same time stabilizing the protein structure. As a result, the accumulation of the enzyme increases to levels significantly higher than the LPS control [47]. To confirm this possibility, it would be necessary to perform further experiments to explore whether F5 and F6 could inhibit COX-2 activity and consequent prostaglandin synthesis.

The capacity of phlorotannin extracts of different seaweeds, as well as of purified phlorotannin fractions and/or compounds to modulate the expression of pro-inflammatory markers, such as iNOS, COX-2, interleukins, and several others, has been largely described [26,28,48]. Studies focusing on phlorotannins from Fucales, however, are much less numerous. Bahar et al. [49], reported that the treatment of ex vivo porcine colonic tissue either with *Ascophyllum nodosum* 80% ethanol extract or *Fucus serratus* water extract significantly inhibited the expression of the genes *IL-8*, *IL-6* and *TNFA,* encoding for the cytokines IL- 8, IL-6 and TNF-α, respectively. Further studies from this research group revealed that both *Ascophyllum nodosum* 80% ethanol extract and *F. vesiculosus* water extract inhibited, more than two-fold, the expression of several cytokines, chemokines, cell adhesion molecules, toll-like receptors (TLR) and other pro-inflammatory mediators in LPS-stimulated porcine colonic tissue [44,50]. In Raw 264.7 macrophages, the treatment with a *F. distichus* purified fraction rich in fucophlorethol oligomers potently inhibited the expression of genes encoding for iNOS, COX-2, TLR4 and 9, IL-1β, IL-6 and IL-17, TNF-α and other pro-inflammatory mediators [41]. However, to the best of our knowledge, such studies have not been carried out yet for phlorotannin purified fractions from *F. vesiculosus*.

#### 2.2.3. Effects on the NF-κB Signaling Pathway

According to the previous results, F2 exhibited the best overall inhibitory effect, being the only sample that significantly decreased the expression of all the three pro-inflammatory proteins analyzed. This was followed by CRD and F3, which markedly inhibited the expression of pro-IL-1β and iNOS as well. It is worth noting that, although not statistically significant, the expression of COX-2 on cells treated with these two samples was still halved compared to LPS-stimulated cells. Note that the expression of these three markers are mediated by several transcription factors from which NF-κB is one of the most important. In fact, a strict relation between iNOS expression and NF-κB transcriptional activity in the presence of LPS has been well established [51], thus the strong inhibitions these three samples displayed against iNOS indirectly indicates that NF-κB transcriptional activity was affected as well. To further evaluate whether CRD, F2 and F3 could interfere with NF-κB activity, the samples were tested for their capacity to inhibit the phosphorylation and degradation of IκBα, and consequent translocation of NF-κB to the nucleus.

For that, in order to determine the peak of phosphorylation and degradation of IκBα, a time course was primarily carried out with LPS-stimulated cells at distinct time points (5, 15, 25, 30, 40, 60 and 70 min). Since the peak of phosphorylation and degradation of IκBα occurred at 15 and 25 min, respectively (data not shown), these were the time points selected for testing the samples.

**Figure 3:**
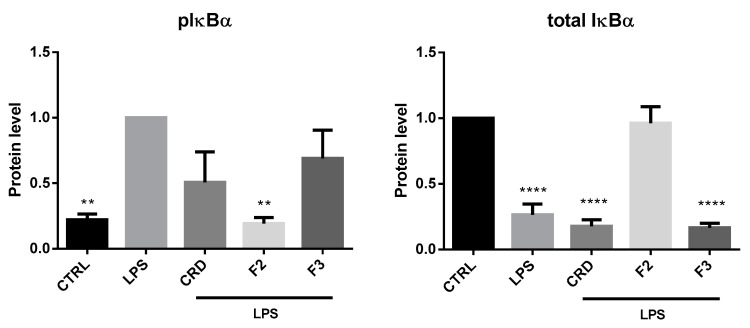
Effects of crude extract, F2 and F3, on the activation of the NF-κB signaling in LPS-stimulated Raw 264.7 cells after 15 min (*p*IκBα) and 25 min (IκBα) of incubation. The immunoblots presented are representative of 3 independent blots. ** *p* < 0.01 and **** *p* < 0.0001 indicate significantly differences from the positive control (with LPS) for *p*IκBα and negative control (CTRL, without LPS) for IκBα, as determined by one-way ANOVA followed by Dunnett’s post hoc test. CRD—Crude extract.

As depicted in Figure 3, the presence of F2 completely abrogated the effects of LPS, maintaining the levels of phosphorylated and total IκBα at the basal levels. As this protein is responsible for maintaining NF-κB sequestered and inactive in the cytoplasm, the inhibition of its phosphorylation and degradation indirectly indicates that the NF-κB translocation to the nucleus was blocked and, consequently, that its transcriptional activity was inhibited. By contrast, the treatment with both CRD and F3 did not significantly affect the phosphorylation of IκBα, although a slight decreasing tendency could be noted. Nevertheless, none of the these two samples were able to prevent the decrease in the total IκBα levels, which means that this protein was still being degraded by the proteasome, and therefore the NF-κB was being freed to translocate to the nucleus.

The involvement of phlorotannins in the activation of NF-κB has been already described by several authors. However, the majority of these works were performed with extracts and/or compounds typical from the genus *Ecklonia.* Indeed, Wei et al. [52] observed that the treatment of LPS-stimulated macrophages with an ethyl acetate fraction obtained from the 95% ethanol extract of *Ecklonia stolonifera* was capable of suppressing NF-κB transcriptional activation not only by preventing IκBα phosphorylation and degradation but also through the inhibition of its translocation to the nucleus. These findings corroborate those previously described by Lee et al. [20], who also reported similar results using a crude ethanol extract of the same species. In turn, an *E. cava* 95% ethanolic extract was shown to suppress the NF-κB translocation and DNA-binding in LPS-stimulated murine BV2 microglia via the inhibition of IκBα degradation [53], while a phlorotannin-rich commercial *E. cava* extract was shown to block the LPS-induced septic shock in C57BL/6 mice through the suppression of the NIK/TAK1/IKK/IκB/NF-κB pathway [54]. Some studies also evidenced promising anti-inflammatory effects through the suppression of NF-κB in different cell lines, for phlorotannin extracts and/or compounds of species from *Eisenia* [55,56,57], *Sargassum* [57,58,59] and other genera [26,44,48,57]. On the other hand, very few studies have focused on the effects of *Fucus* phlorotannins in NF-κB transcriptional activity. In fact, to our knowledge, only one study has shown that *F. vesiculosus* cold-water extract was capable of inhibiting the NF-κB gene expression in ex vivo porcine colonic tissue [50]. Therefore, the present work provides new insights into the potential of *F. vesiculosus* to modulate inflammation, showing that this seaweed may also be a source of relevant phlorotannin compounds capable of suppressing the NF-κB transcriptional activity through the inhibition of the phosphorylation and proteosomal degradation of the IκBα.

### 2.3. Characterization of F2

**Figure 4:**
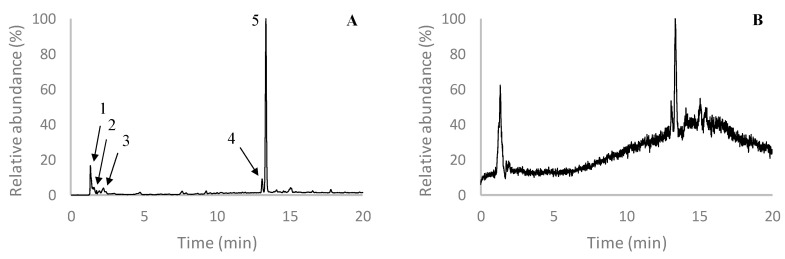
Chromatographic profile at 280 nm (**A**) and total ion chromatogram (**B**) of F2. Peaks marked with numbers correspond to the tentatively identified compounds represented in Table 2.

In order to elucidate the compounds present in the most active *F. vesiculosus* fraction—i.e., F2—an UHPLC-MS analysis was carried out. Overall, the chromatogram of this fraction (Figure 4) was characterized by the presence of a major peak eluting at 13.4 min that showed a deprotonated molecular ion at *m/z* 507. Although, to our knowledge, no phlorotannin with such molecular weight has been described before, the MS/MS spectrum of this compound revealed a base peak at *m/z* 489, corresponding to the loss of water, which is a common characteristic of phlorotannins, and two major peaks at *m/z* 277 and 229, the former indicating the loss of fucol moiety ([M−H−230]^−^) and the later corresponding to the fucol moiety itself. Additionally, other minor product ions also evidenced a fragmentation pattern coherent with those previously described for phlorotannins. This is the case of the product ions at *m/z* 463, which results from the elimination of ethanal (44 Da) as a consequence of the internal cleavage of benzene ring structures, at *m/z* 445, corresponding to the loss of 44 Da plus an additional water molecule, and at *m/z* 479, corresponding to the loss of CO or ethylene (28 Da), also resultant from cross ring cleavage [14,60]. The product ion at *m/z* 245 is indicative of a phloroglucinol dimer, while the one at *m/z* 261 (-246 Da) indicates the loss of a phloroglucinol dimer [14,61]. Therefore, although the precise identification of this compound was not achieved, this evidence allows us to conclude that it may correspond to a phlorotannin derivative.

Interestingly, peak 4, eluting at 13.1 min, showed a [M − H]^−^ at *m/z* 587 and further fragmented into the main product ion at *m/z* 507, also yielding other minor product ions that were common with those observed in the MS/MS spectrum of the compound described above—namely at *m/z* 277 and 229. Due to these similarities, it is possible that this compound may not only be a phlorotannin derivative but also a derivative of the compound with the [M − H]^−^ at *m/z* 507, and, even though it has not been identified yet, previous studies have already reported its presence in extracts of *F. vesiculosus*. This is the case of our previous work conducted with *F. vesiculosus* EtOAc purified extract, in which the deprotonated molecular ion at *m/z* 587 originated the same major product ion at *m/z* 507 [14]. Other authors also reported the presence of a compound with the same [M − H]^−^ and MS^2^ base peak in a water extract of *F. vesiculosus* from Camariñas, Spain, although they were also unable to identify it [62].

Apart from these two major compounds, eluting in peaks 4 and 5, others were detected in F2 with lower intensities, including two isomers with [M − H]^−^ at *m/z* 497 and two other compounds with [M−H]^−^ at *m/z* 511 and 529. The first two compounds eluted at 1.4 and 2.0 min (peak 1 and 2) and correspond to two isomers of a phloroglucinol tetramer, both showing MS/MS spectra coherent with previous works [14,60]. Although it is not possible to ensure their exact structural arrangements, the absence of phloroglucinol moieties in the MS^2^ spectrum of the isomer eluting at 1.4 min suggest it might correspond to a tetrafucol—i.e., a tetramer composed exclusively of C-C linked phloroglucinols. In turn, the presence of the product ions at *m/z* 371 (-126 Da) and 353 (-126-18 Da) on the MS^2^ spectrum of the isomer eluting at 2.0 min indicates the loss of a phloroglucinol moiety and a phloroglucinol combined with water, respectively, suggesting that this compound contains at least one C-O-C linkage [14]. The compound with [M−H]^−^ at *m/z* 529 (eluting at 2.4 min, peak 3) also showed an MS spectrum coherent with that of a phloroglucinol tetramer, although containing two additional OH groups, which is a characteristic feature of fuhalols. Therefore, based on these findings, as well as on literature data, this compound was attributed to hydroxytetrafuhalol [63]. Co-eluting at the same retention time, the [M−H]^−^ at *m/z* 511 exhibited a fragmentation pattern with losses that typically occur in phlorotannins, including -18 Da (water), -44 Da (ethenal) and -126 Da (phloroglucinol unit), as well as their combinations (Table 2). Moreover, the product ion at *m/z* 493 appears as the main product ion of this compound, which is in agreement with what Mezghani et al. [64] previously described for diphlorethohydroxycarmalol, although their work was carried out using extracts of *Ulva rigida*, a species belonging to the Chlorophyta, which contradicts the supposed exclusivity of phlorotannins to the Phaeophyta.

Notably, the presences of phloroglucinol tetramers and hydroxytetrafuhalol have already been described in *F. vesiculosus* before, including in a previous work from our research group [14,15,62]. However, until now, with exception of the work reported by Mezghani et al. [64], the appearance of diphlorethohydroxycarmalol has been exclusive to the species *Ishige okamurae* [65,66]. Therefore, this is the first work reporting the presence of diphlorethohydroxycarmalol in a brown algae species other than *Ishige okamurae*, although further spectroscopic analysis would be necessary to ensure this hypothesis.

## 3. Materials and Methods

### 3.1. Chemicals

Grounded *F. vesiculosus* samples from July 2017 were purchased from Algaplus Lda (production site located at Ria de Aveiro coastal lagoon, Northern Portugal, 40^o^36′43” N, 8^o^40′43” W), an enterprise dedicated to the production of edible seaweeds in an integrated multi-trophic aquaculture (IMTA) system. Acetone, ethanol, methanol, *n*-hexane, ethyl acetate, acetonitrile, dimethyl sulfoxide and hydrochloric acid were acquired from Fisher (Pittsburgh, PA, USA). Fluorescein, 2,2′-azobis(2-amidinopropane) di-hydrochloride (AAPH), sodium nitroprusside and sulfanilamide were purchased from Acros Organics (Hampton, NH, USA). Formic acid, phosphate buffer saline (PBS) reagents (sodium salt, sodium chloride, potassium chloride, disodium hydrogen phosphate and potassium dihydrogen phosphate), trolox, ascorbic acid, gallic acid, β-nicotinamide adenine dinucleotide, reduced nicotinamide adenine dinucleotide disodium salt hydrate (NADH), nitro blue tetrazolium (NBT), phenazine methosulfate (PMS), xanthine oxidase, allopurinol, Dulbecco’s modified Eagle’s medium (DMEM), Tween^®^ 20, penicillin G sodium salt, streptomycin sulfate salt, sodium bicarbonate, D-glucose, lipopolysaccharide (LPS) from Escherichia coli—serotype 026:B6—and anti-β-actin antibody were purchased from Sigma (St. Louis, MO, USA). Anti-COX-2, anti-pro-IL-1β and alkaline phosphatase-conjugated anti-mouse antibodies were purchased from Abcam (Cambridge, UK). Anti-IκBα and anti-*p*IκBα (Serine 32/36) antibodies were purchased from Cell Signaling Technologies (Danvers, MA, USA). Anti-iNOS antibody was acquired from R&D Systems (Minneapolis, MN, USA) and alkaline phosphatase-conjugated anti-rabbit antibody from Santa Cruz Biotechnology, Inc. (Santa Cruz, Dallas, TX, USA). Fetal bovine serum (FBS) was purchased from Gibco (Paisley, UK), xanthine from AlfaAesar (Ward Hill, MA, USA), and sodium di-hydrogen phosphate was acquired from Panreac (Barcelona, Spain). All reagents were of analytical grade or of the highest available purity.

### 3.2. Extraction and Purification of Phlorotannins from Fucus vesiculosus

**Figure 5:**
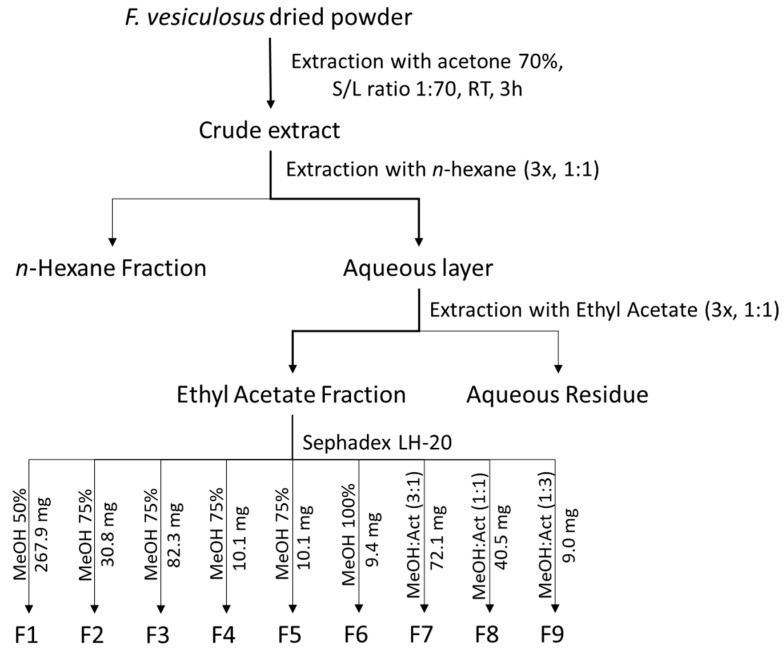
Flowchart for extraction and fractionation of phlorotannins from *F. vesiculosus*. F1 to F9 represent nine subfractions obtained from the Sephadex LH-20 column chromatography with a solvent system of decreasing polarity. S/L—solid/liquid ratio (g/mL), RT—room temperature, MeOH—methanol, Act—acetone.

Extraction and solvent partitioning were performed as depicted in Figure 5, following the method described by Catarino et al. [14]. Briefly, 100 g of dried algal powder were dispersed in 7000 mL of 70% acetone solution for 3 h at room temperature (RT) under constant agitation and filtered through a G4 glass filter, yielding a crude extract (CRD) with a total phlorotannin content of 10.7 ± 1.5 mg PGE/g extract. Afterwards, the extract was defatted with *n*-hexane (1:1 *v*/*v*, 3 times) and the aqueous layer was extracted with ethyl acetate (1:1 *v*/*v*, 3 times), yielding an ethyl acetate-soluble fraction (EtOAc) with a phlorotannin content of 17.1 ± 1.5 mg PGE/g extract. In order to obtain sub-fractions of different molecular weights, this EtOAc-soluble fraction was further submitted to gel filtration on a Sephadex LH-20 column according to the procedure reported by Wang et al. [15], using solvents of decreasing polarity eluting stepwise, namely aqueous methanol 50% (*v*/*v*), aqueous methanol 75% (*v*/*v*), pure methanol, methanol and acetone 3:1 (*v*/*v*), methanol and acetone 1:1 (*v/v*) and finally methanol and acetone 1:3 (*v*/*v*). From this gel filtration, nine fractions were recovered (F1—267.9 mg, F2—30.8 mg, F3—82.3 mg, F4—10.1 mg, F5—10.1 mg, F6—9.4 mg, F7—72.1 mg, F8—40.5 mg and F9—9.0 mg), and the solvents were evaporated under reduced pressure prior to lyophilization and storage at −20 °C.

### 3.3. Antioxidant Experiments

#### 3.3.1. ORAC

The ORAC assay was performed according to the method previously described by Catarino et al. [32]. In a 96-well plate, 150 µL of fluorescein (10 nM), diluted from the stock solution of 250 µM, with 75 mM sodium dihydrogen phosphate buffer (pH 7.4), were placed together with 25 µL of different trolox concentrations (3.13–25 µM). The same process was repeated for the samples. After 10 min incubation at 37 °C, 25 µL of AAPH (153 mM) solution was added to each well and the plate was immediately placed in the plate reader (Biotek, Vienna, Austria), for monitoring the fluorescence (excitation 485 nm and emission at 528 nm) every min over 60 min at 37 °C. Using the calibration curve of trolox, the ORAC value was calculated and expressed as μmol of trolox equivalents (TE) per g of extract.

#### 3.3.2. Superoxide Anion (O_2_^•−^) Scavenging Assay

The O_2_^•−^ scavenging method was carried out according to the method described by Pereira et al. [67]. Briefly, in a 96-well plate, 75 µL of six different sample concentrations (0.0–2.0 mg/mL) were mixed with 100 µL of β-NADH (300 µM), 75 µL of NBT (200 µM) and 50 µL of PMS (15 µM). After 5 min, the absorbances at 560 nm were recorded and the inhibition calculated as the concentration capable of scavenging 50% of O_2_^•−^ (EC_50_). Gallic acid was used as the reference compound

#### 3.3.3. Xanthine Oxidase Assay

Inhibition of xanthine oxidase activity was carried out following the method described by Pereira et al. [67], with slight modifications. Briefly, in a 96-well plate, 40 µL of sample (0–2 mg/mL) was mixed with 45 µL of sodium dihydrogen phosphate buffer (100 mM, pH 7.5) and 40 µL of enzyme (5 mU/mL). After 5 min incubation at 25 °C, the reaction was initiated by adding 125 µL of xanthine (0.1 mM dissolved in buffer) and the absorbance at 295 nm was measured every 45 s over 10 min at 25 °C. The inhibitory capacity was then calculated as the concentration of the sample capable of inhibiting 50% of the enzyme’s activity. Allopurinol was used as a reference inhibitor.

#### 3.3.4. Nitric Oxide (NO^•^) Assay

The NO^•^ scavenging method was adapted from Pereira et al. [67]. Briefly, 100 µL of six different sample concentrations (0–1 mg/mL) were mixed with 100 µL of sodium nitroprusside (3.33 mM in 100 mM sodium phosphate buffer pH 7.4) and incubated for 15 min under a fluorescent lamp (Tryun 26 W). Next, 100 µL of Griess reagent (0.5% sulfanilamide and 0.05% N-(1-naphthyl)-ethylenediamine dihydrochloride in 2.5% H_3_PO_4_) were added to the mixture, which was incubated for another 10 min at RT in the dark. The absorbance was then measured at 562 nm, and the NO^•^ scavenging capacity was calculated as the concentration of sample capable of scavenging 50% of the radical. Ascorbic acid (4.2–66.7 µg/mL) was used as the reference compound.

### 3.4. Anti-Inflammatory Experiments

#### 3.4.1. Cell Culture

Raw 264.7 cells, a mouse leukemic monocyte macrophage cell line (ATCC TIB-71), were cultured in DMEM media supplemented with 10% inactivated fetal bovine serum, 100 U/mL penicillin and 100 μg/mL streptomycin, at 37 °C in a humidified atmosphere of 95% air and 5% CO_2_. Along the experiments, cells were monitored by microscopy in order to detect any morphological change.

#### 3.4.2. Assessment of Cell Viability

The effect of each sample on cell viability/metabolic activity was evaluated according to the resazurin assay previously described [68]. For this assay, cells (6 × 10^4^ cells/well) were plated in 96-well plates and allowed to stabilize overnight. Cells were then exposed to serial concentrations of each sample reconstituted in DMEM with 0.5% DMSO, which has been previously shown to have minimal impact on Raw 264.7 viability [69]. After 24h incubation, resazurin (50 μM) was added to the cells 3 h prior recording absorbance at 570 nm, with a reference wavelength of 620 nm, using a standard spectrophotometer. The results were expressed relative to untreated cells viability/metabolic capacity.

#### 3.4.3. Inhibition of LPS-Stimulated NO^•^

The effect of each sample on the nitrite production in LPS-stimulated Raw 264.7 cells was measured using a colorimetric reaction with Griess reagent as described elsewhere [68]. For that, cells were plated and treated, as described above in 3.4.2 section. The LPS stimulus (50 ng/mL) was added 1 h post samples treatment, and 24 h later, the cell-free supernatants were collected and diluted with equal volumes of Griess reagent in the dark. After 30 min, the absorbance was registered in an automatic microplate reader at 550 nm. The extent of the inhibition of nitrite production was evaluated based on the comparison with untreated cells.

#### 3.4.4. Preparation of Total protein Extracts and Western Blotting

Raw 264.7 cells (1.2 × 10^6^ cells/well) were seeded in 12-well plates and allowed to stabilize overnight. Cells were either maintained in culture medium (negative control), or pre-incubated with the highest concentrations of samples that inhibited NO^•^ generation and without decreasing cell viability below 90%, for 1 h before the addition of LPS (50 ng/mL) for different time points, according to the proteins studied. The protein extraction was then carried out as descried before [32]. Western blotting was subsequently performed for evaluation of protein levels of iNOS, COX-2 and pro-IL-1β. Briefly, equivalent amounts of protein were electrophoretically separated on a 10% (v/v) sodium dodecyl sulfate polyacrylamide gel and transferred to a PVDF membrane. The membranes were blocked with 5% (*w*/*v*) fat-free dry milk in TBS-T, for 1 h at RT. Blots were then incubated overnight at 4 °C with the primary antibody against: COX-2 (1:10,000), iNOS (1:500), pro-IL-1β (1:500), IκBα (1:1000) and *p*IκBα (1:1000). After washing 3 times during 10 min with TBS-T, the membranes were further incubated for 1 h at RT with alkaline phosphatase-conjugated anti-mouse (for iNOS and *p*IκBα) and anti-rabbit (for COX-2, pro-IL-1β and IκBα) secondary antibodies. The detection of the immune complexes was followed by scanning for blue excited fluorescence on the Typhoon imager (GE Healthcare) after 5 min of membrane exposure to the ECF reagent. The generated signals were analyzed using the ImageQuant TL software. Thereafter, the membranes were stripped, and the same process was repeated with mouse anti-β-actin (1:5000) antibody, which was used as a loading control and for the normalization of the results. The results exhibited correspond to the ratio of target protein/β-actin.

### 3.5. UHPLC-DAD-ESI/MS Analysis

Chromatographic analysis was carried out as reported in Catarino et al.’s [14] work for the *F. vesiculosus* phlorotannin fraction that showed the highest anti-inflammatory activity in an Ultimate 3000 (Dionex Co., San Jose, CA, USA), an apparatus consisting of an autosampler/injector, a binary pump, a column compartment and an ultimate 3000 Diode Array Detector (Dionex Co., San Jose, CA, USA), coupled to a Thermo LTQ XL (Thermo Scientific, San Jose, CA, USA) ion trap mass spectrometer equipped with an ESI source. The LC separation was carried out in a Hypersil Gold (ThermoScientific, San Jose, CA, USA) C18 column (100 mm length; 2.1 mm i.d.; 1.9 µm particle diameter, end-capped) maintained at 30 ◦C and a binary solvent system composed of (A) acetonitrile and (B) 0.1% of formic acid (*v*/*v*). The solvent gradient started with 5–40% of solvent (A) over 14.72 min, from 40–100% over 1.91 min, remaining at 100% for 2.19 more min before returning to the initial conditions. The flow rate was 0.2 mL/min and UV–Vis spectral data for all peaks were accumulated in the range of 200–700 nm while the chromatographic profiles were recorded at 280 nm. Control and data acquisition of MS were carried out with the Thermo Xcalibur Qual Browser data system (ThermoScientific, San Jose, CA, USA). Nitrogen above 99% purity was used, and the gas pressure was 520 kPa (75 psi). The instrument was operated in negative mode with the ESI needle voltage set at 5.00 kV and an ESI capillary temperature at 275 °C. The full scan covered the mass range from *m*/*z* 100 to 2000. CID–MS/MS experiments were performed for precursor ions using helium as the collision gas with a collision energy of 25–35 arbitrary units. All solvents used were of LC-MS grade

### 3.6. Statistical Analysis

Data were expressed as mean ± standard deviation (SD) of three similar and independent experiments in the antioxidant experiments, whereas for the cellular experiments, data were expressed as mean ± standard error of the mean (SEM) of three similar and independent experiments. One-way ANOVA followed by Tukey’s post hoc test was performed for all the antioxidant assays with the exception of ORAC, for which a two-tailed unpaired *t*-test was used. In turn, one-way ANOVA followed by Dunnet’s post hoc test was performed for the experiments carried out on cells. The statistical tests were applied using GraphPad Prism, version 7.00 (GraphPad Software, San Diego, CA, California) and the significance level was *p* < 0.05.

## 4. Conclusions

This study provides evidence of the antioxidant and anti-inflammatory activities of *Fucus vesiculosus* phlorotannin extract and subsequent purified fractions, as reflected by their ability to scavenge chemically-generated ROS and to inhibit inflammatory response on LPS-induced macrophages. Both crude extract and ethyl acetate fraction displayed good scavenging activity against the different radicals tested, especially NO^•^, a free radical that plays a pivotal role in the signaling and pathogenesis of inflammation, indicating that these extracts may not only exhibit good antioxidant potential but also a possible anti-inflammatory capacity. This hypothesis was further confirmed with biological experiments using LPS-stimulated Raw 264.7 cells. At 100 μg/mL, both phlorotannin-rich samples decreased the NO^•^ production to approximately 85% of the untreated cells. The inhibitory effects observed for the subsequent fractions further allowed us to demonstrate a possible relation between phlorotannins’ complexity and anti-inflammatory activity, with lower molecular weights evidencing stronger effects. The expression of iNOS was the most affected, being susceptible to every sample, followed by IL-1β, inhibited by all samples except F1 and F5, and COX-2 which was only inhibited by F2. The potent inhibitory effects observed for this fraction were finally demonstrated to be resultant from its capacity to inhibit IκBα phosphorylation and degradation, which translates to the blockage of the NF-κB signaling pathway. This fraction was characterized by the presence of some minor phlorotannin tetramers and a major compound with [M − H]^−^ at *m*/*z* 507 which could be attributed to a phlorotannin derivative based on the fragmentation products present in its MS/MS spectrum. Overall, this study contributes for a better understanding of the mechanisms behind the anti-inflammatory properties of *F. vesiculosus* phlorotannins extract and purified fractions. However, it must be considered that natural products are always susceptible to great variability. Therefore, further research is necessary not only to verify the reproducibility of these results but also to elucidate the structural features of the compound detected in F2 and evaluate whether it is the responsible for the remarkable anti-inflammatory activity displayed by this fraction.

## Figures and Tables

**Table 1:** Antioxidant activities of *F. vesiculosus* crude extract, ethyl acetate fraction and the respective reference compounds.

Sample	RCOO^•^ (μmol TE/g ext) ^(1)^	O_2_^•−^ (IC_50_ μg/mL) ^(2)^	NO^•^ (IC_50_ μg/mL) ^(3)^	XO (IC_50_ μg/mL) ^(4)^
CRD	3395.04 ± 211.4 ^a^	98.7 ± 11.1^a^	75.2 ± 5.1^a^	2.8 ± 0.4^a^
EtOAc	2986.04 ± 338.7 ^b^	268.0 ± 20.1^b^	235.9 ± 19.5^b^	1.2 ± 0.2^b^
Standard	-	7.8 ± 0.5^c^	212.1 ± 9.7^b^	0.1 ± 0.01^c^

^(1)^ TE—Trolox equivalent, ^(2)^ Standard compound for O_2_^•−^ is gallic acid, ^(3)^ Standard compound for NO^•^ is ascorbic acid, ^(4)^ Standard compound for xanthine oxidase (XO) is allopurinol. CRD – Crude extract, EtOAc—Ethyl acetate fraction. IC_50_ value was determined as the concentration at which O_2_^•−^, NO^•^ and XO activity were reduced by 50%. Mean values ± SD; statistical analysis was performed by two-tailed unpaired *t*-test for ORAC, and one-way ANOVA followed by Tukey’s test for the remaining assays. In each column, different letters mean significant differences (*p* < 0.05).

**Table 2:** Tentative assignment of the compounds detected in the F2 analyzed by UHPLC-ESI-MS/MS.

Peak	RT (min)	[M − H]^−^ (*m/z*)	MS/MS Ions (-loss)*	Tentative Assignment
1	1.4	497	479 (-18), 331 (-PGU-44), 461 (-18-18), 435 (-44-18), 453 (-44), 413 (-84), 395 (-84-18), 347 (-150), 305 (-192), 165(-2PGU-84), 315 (-PGU-44-18)	Tetrafucol
2	2.0	497	479 (-18), 331 (-PGU-44), 461 (-18-18), 435 (-44-18), 453 (-44), 395 (-84-18), 305 (-192), 165 (-2PGU-84), 315 (-PGU-44-18), 371 (-PGU), 353 (-PGU-18)	Fucophlorethol
3	2.4	511	493 (-18), 449 (-44-18), 475 (-18-18), 467 (-44), 439 (-72), 411 (-84-16), 405 (-106), 345 (-PGU-44), 301 (-210), 331 (-180), 347 (-164), 385 (-PGU), 395 (-98-18), 369 (-PGU-16), 351 (-PGU-84), 329 (-PGU-44-18), 313 (-PGU-72)	Diphlorethohydroxycarmalol
		529	511 (-18), 493 (-18-18), 411 (-84-16-18), 467 (-44), 429 (-84-16), 439 (-90), 347 (-PGU-16-44)	Hydroxytetrafuhalol
4	13.1	587	507 (-80), 523 (-64), 229 (-PGU-108-80-44), 277 (-230-80), 489 (-80-18), 383 (-PGU-80), 399 (-108-80), 463 (-PGU), 275 (-232-80), 569 (-18)	Phlorotannin derivative
5	13.4	507	489 (-18), 277 (-230), 229 (-PGU-108-44), 461 (-46), 463 (-44), 479 (-28), 445 (-44-18), 275 (-232), 261 (-246), 245 (-262), 421 (-86), 297 (-PGU-84)	Phlorotannin derivative

* Product ions are arranged in descending order of relative abundance. PGU—phloroglucinol unit (-126/124).
